# Emotional stress during the COVID-19 lockdown: how negative X/Twitter posts correlated with changes in the brain's fear network

**DOI:** 10.3389/fnume.2025.1575026

**Published:** 2025-06-10

**Authors:** Eric Guedj, Jacques-Yves Campion, Tatiana Horowitz, Fanny Barthélémy, Stéphanie Khalfa, Wissam El-Hage

**Affiliations:** ^1^ Aix Marseille Univ, APHM, CNRS, Centrale Marseille, Institut Fresnel, Timone Hospital, CERIMED, Nuclear Medicine Department, Marseille, France; ^2^ Université de Tours, INSERM, Imaging Brain & Neuropsychiatry IBraiN U1253, Tours, France; ^3^ Department of Psychiatry, University of Pittsburgh, Pittsburgh, PA, United States; ^4^ Aix Marseille Univ, APHM, CNR, Centre de Recherches en Psychologie et Neurosciences, Marseille, France; ^5^ CHRU de Tours, Regional Trauma Center CRP-CVL, Tours, France

**Keywords:** COVID-19, SARS-CoV-2, lockdown, [18F]FDG PET, brain metabolism, Twitter, media, communication

## Abstract

**Introduction:**

The COVID–19 pandemic has profoundly affected mental health, with lockdown periods particularly exacerbating negative emotions such as fear, sadness, and uncertainty. This study examines brain metabolic changes associated with the psychological context of the first French COVID–19 lockdown in vulnerable individuals.

**Methods:**

As a proxy measure of the psychological context, we used a composite negative–emotion score derived from an open–source X/Twitter dataset (“The First French COVID–19 Lockdown Twitter Dataset”), designed to capture public sentiment over the 55–day lockdown. This score was day–by–day correlated with whole–brain voxel–based [18F]FDG PET imaging in 95 patients with neurological conditions, using statistical parametric mapping (SPM) (*p*–voxel < 0.001, *k* > 108).

**Results:**

A significant negative correlation was found between daily negative–emotion scores and metabolism in the right ventromedial prefrontal cortex (vmPFC) and anterior cingulate cortex (ACC), key regions of the brain's fear circuit. Inter–regional correlation analysis (IRCA) of metabolic connectivity from the right vmPFC/ACC further revealed a right limbic–dominant network including the amygdala, hippocampus, thalamus, and basal ganglia.

**Discussion:**

These findings highlight the sensitivity of the right vmPFC/ACC to societal emotional stressors, suggesting a potential cerebral substrate for the increase in psychological and psychiatric disorders observed during the pandemic. Further research is needed to validate these results in larger populations and to explore their longitudinal implications, to better understand the neurological impact of collective stress.

## Introduction

Pandemics have consistently demonstrated significant and lasting negative impacts on mental health (1). During and after the COVID-19 pandemic, widespread issues such as anxiety, mood disorders, and posttraumatic stress disorder (PTSD) were frequently reported (2), particularly among individuals who experienced severe illness, the loss of loved ones, or other pandemic-related stressors. Additionally, rising substance use and sleep disturbances have emerged as major concerns. Isolation, uncertainty, and fear have been identified as key psychological mechanisms underlying these impacts, with the lockdown period particularly exacerbating these effects (1, 3–5). The psychological burden was further compounded by physical deconditioning during prolonged confinement (1, 5, 6). Globally, more than 3 billion people were confined to their homes to curb the spread of the virus (1), underscoring the unprecedented scale of this intervention. In France, a national lockdown was enforced between March 17 and May 11, 2020, during which only essential workers were permitted to leave for work, outdoor physical activity was limited to within one kilometer of residence, and all non-urgent medical procedures were suspended (7). Meanwhile, psychiatric symptoms have also been reported in patients with long COVID, mostly associated with neuroinflammation and microglial activation, underscoring the multifaceted impact of the COVID-19 pandemic on the brain (8–13).

Beyond brain disorders, enviromics — as a complement to genomics — highlights how environmental and societal factors can modulate brain function and structure, and interact with biological vulnerability (14). This conceptual approach of population neuroscience is increasingly applied in neuroimaging to understand how collective experiences influence large-scale brain organization (6, 14). In a previous metabolic PET study conducted during the French lockdown, we demonstrated the impact of physical deconditioning on sensorimotor networks in vulnerable patients with neurological diseases (6). Building on this work, we now aim to explore the mental impact of the lockdown on brain PET metabolism, applying the same methodological approach of statistical parametric mapping (SPM) to the same patient population. As a proxy for the psychological context of this period, we leveraged a composite negative-emotion score from an open-source X/Twitter dataset (“The First French COVID-19 Lockdown Twitter Dataset”) (15). This dataset, previously analyzed and published by another research group, was designed to capture global sentiment during the 55-day lockdown in France (15). Whole-brain voxel-based PET analysis was used to correlate [18F]FDG metabolism with the negative-emotion score from the day of the exam (6), and to identify associated brain networks by inter-regional correlation analysis (IRCA) (16–18). These findings were then compared with previous PET studies on physical deconditioning during the lockdown (6) and long COVID (9), providing a comprehensive view the neural effects of collective stress.

## Materials and methods

### PET procedure

This research retrospectively utilized PET data collected in clinical settings during the 55-day national lockdown in France, from March 17 to May 11, 2020 (19). Patients examined during this period were systematically tested for COVID-19 before undergoing the PET scan. Those who tested positive had their examination postponed until the active phase of the disease had resolved. Brain PET-CT scans were acquired using the same GE Discovery MI5 PET/CT camera in the Nuclear Medicine Department of Timone Hospital, following international guidelines (20, 21). Imaging was performed with a three-dimensional protocol, consisting of a 15-minute scan conducted 30 min after the intravenous injection of 150 MBq of [^1^⁸F]FDG at rest. Image reconstruction utilized the VPFX protocol, incorporating attenuation correction based on the corresponding CT acquisition. The final reconstructed images had a matrix size of 256 × 256, with a pixel size of 1.3672 × 1.3672 mm, a resolution of 0.7314 pixels per mm, and covered 89 slices.

This study was conducted in compliance with French research guidelines and in absence of patient opposition, received approval from the APHM institutional review board on March 11, 2021, under the reference PADS21-84.

### X/Twitter sentiment analysis

To approximate the psychological context of the French lockdown, we utilized a composite negative-emotion score derived from an open-source X/Twitter dataset, previously published and analyzed by another research group (15). This score was designed to capture global public sentiment during the 55-day lockdown (15). Specifically, it was obtained through sentiment analysis of word-emotion associations using “The First French COVID-19 Lockdown Twitter Dataset” (15), which includes 2,598,249 tweets collected between March 17 and May 11, 2020. After removing duplicate tweets, the dataset was organized chronologically. Tweets were collected using the Twitter REST API and the *rtweet* package in R. Sentiment analysis relied on the NRC emotion lexicon, focusing on negative sentiment categories — fear, anger, disgust, sadness, and surprise/amazement — to compute a negative-emotion score for each individual tweet (22). This score, exclusively positive, reflects the intensity of negative emotional content, with higher values indicating stronger negativity. A daily negative-emotion score was calculated by averaging the individual negative-emotion scores across all tweets published each day.

### Neuroimaging and statistical analyses

Using SPM (Wellcome Department of Cognitive Neurology, University College, London, UK), we analyzed whole-brain voxel-based correlations between the negative-emotion score and [18F]FDG PET images from the same day. This analysis included spatial normalization, smoothing (8 mm FWHM), and global proportional scaling. A relative similar SPM methodology has been previously applied to investigate relationships between gray matter volume and specific skills or behaviors, such in London taxi drivers using MRI (23), as well as in our earlier work on the physical impact of the COVID-19 lockdown (6), and more broadly within the scientific framework of “enviromics” in population neuroscience (14). The voxel threshold for significance was set at *p* < 0.001, with cluster size correction using Monte Carlo simulation (minimum 108 voxels).

To control for potential confounding factors, we included age, sex, presence of focal morphological lesion (yes/no), and the duration of lockdown at the time of the PET exam as covariates in the statistical model. We further explored the effects of these variables using Pearson's correlations and partial correlations, in addition to ANOVA to evaluate the influence of distinct neurological conditions (cognitive/behavioral impairment, glioma, and focal epilepsy).

The identified metabolic cluster was extracted using MarsBaR (http://marsbar.sourceforge.net/) and employed as a seed region for metabolic networks identification by inter-regional correlation analysis (IRCA) (16–18), applying the same statistical threshold (*p* < 0.001; *k* > 108).

The findings were then compared to previously reported brain metabolic profiles associated with physical deconditioning during the lockdown (6) and with long COVID (9), assessing overlap and unique voxel patterns. Anatomical regions were identified using atlas from the WFU PickAtlas (https://www.nitrc.org/projects/wfu_pickatlas/), ensuring precise localization of the implicated areas.

## Results

During the 55-day national lockdown in France, from March 17 to May 11, 2020, 95 adult patients (mean age: 54.3 ± 15.7 years; 59 men; Table 1) underwent [18F]FDG brain PET scans in our Nuclear Medicine Department. These scans were performed for the following clinical indications: cognitive or behavioral impairment (*n* = 49), glioma (*n* = 41), and focal epilepsy (*n* = 5), with 47 cases presenting identified focal morphological lesion (Table 1).

**Table 1:** Demographic and clinical characteristics of patients.

Characteristics	
*N*	95
Age (mean ± SD, min -max)	54.3 ± 15.7 years (19–85)
Male (*n*)	59
Patients with cognitive or behavioral impairment (*n*)	49
Patients with glioma (*n*)	41
Patients with epilepsy (*n*)	5
Patients with lesion (*n*)	47
Composite negative-emotion score (mean ± SD, min–max)	1.11 ± 0.24 (0.44–1.66)

**Figure 1:**
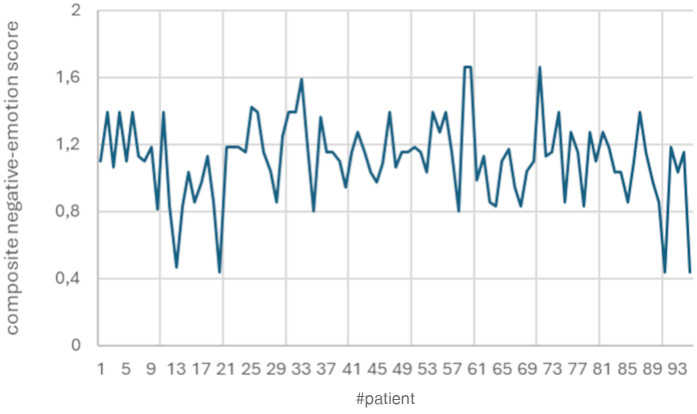
Distribution of composite negative-emotion scores across patients. The mean daily negative-emotion score during the lockdown was 1.11 ± 0.24, ranging from 0.44 to 1.66.

The mean daily negative-emotion score during the lockdown was 1.11 ± 0.24, ranging from 0.44 to 1.66 (Figure 1). A positive correlation was observed between the duration of the lockdown and the daily negative-emotion score (*r* = 0.33, *p* = 0.0011).

**Figure 2:**
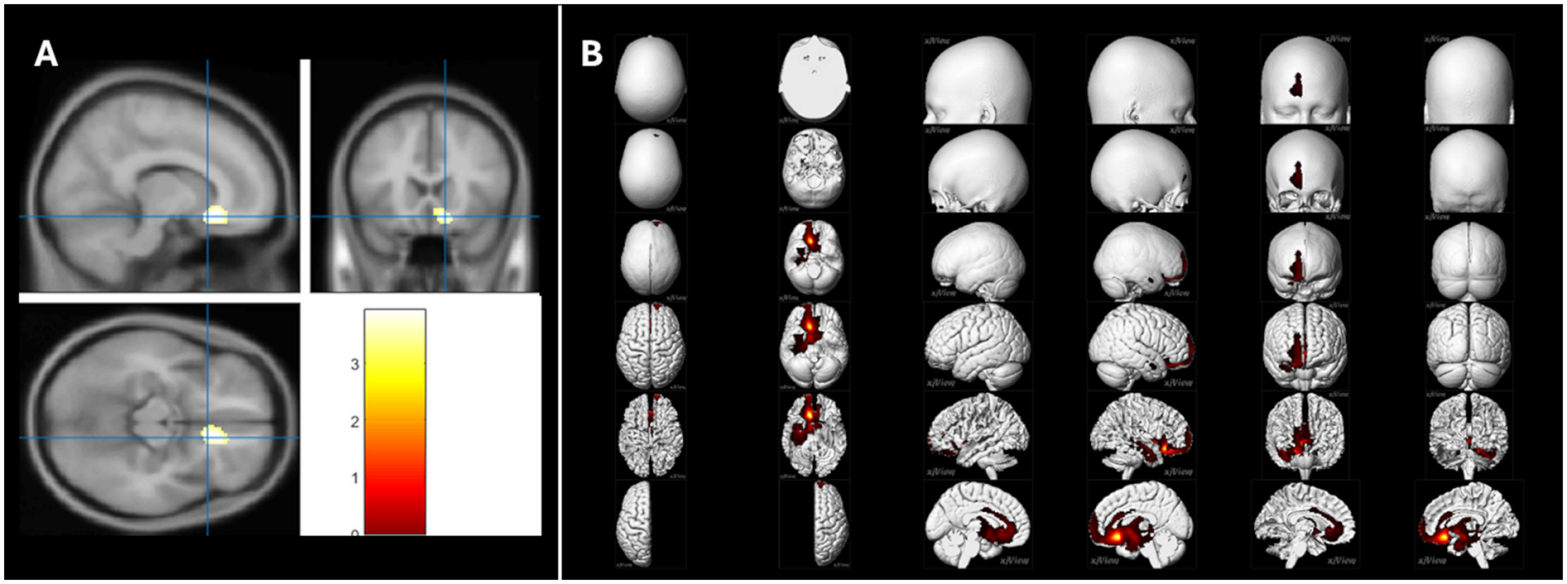
Brain PET T-maps relationships with the daily negative-emotion score. **(A)** During the lockdown period, a negative correlation was observed between the daily negative-emotion score and metabolism in the right ventromedial prefrontal cortex (vmPFC) and anterior cingulate cortex (ACC). Higher negative-emotion scores were associated with lower metabolic activity in these regions (peak *T*-score = 3.93, *p*-voxel < 0.001, *k* = ind; Pearson's correlation: *r* = −0.40, *p* = 0.00007). This correlation remained significant after controlling for age, sex, presence of focal morphological lesion, and duration of the lockdown. **(B)** The metabolic connectivity associated with this right vmPFC/ACC cluster revealed an extensive network predominantly involving the right hemisphere, with a limbic component. This network encompassed fronto-temporal regions, the insula, and the anterior cingulate cortex, as well as the medial temporal lobe structures, including the amygdala, hippocampus, and parahippocampus. Additionally, subcortical regions such as the basal ganglia, thalamus, and midbrain were also involved (peak *T*-score = 60.47, *p*-voxel < 0.001, *k* = 10,201). These findings were independent of age, sex, presence of focal morphological lesion, and duration of the lockdown.

**Figure 3:**
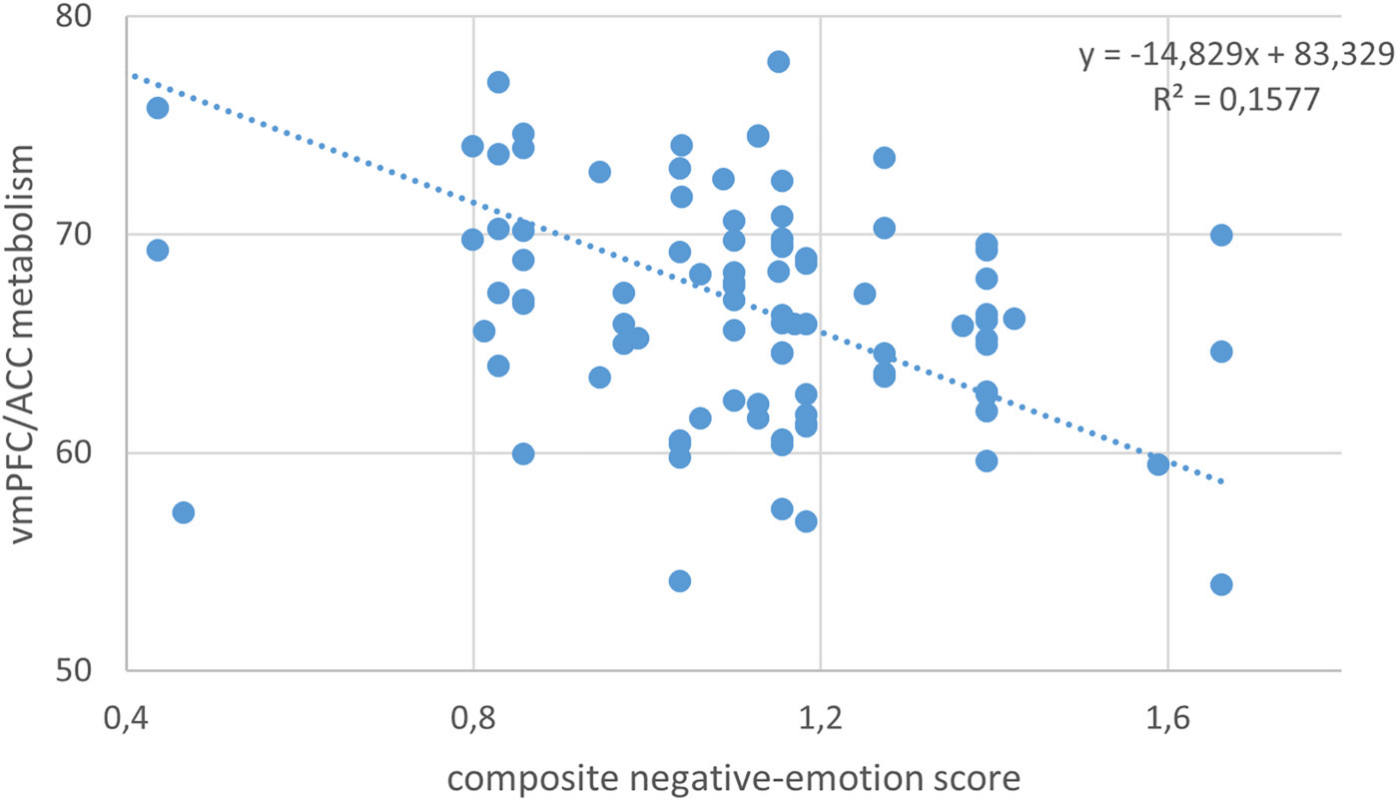
Negative correlation between the daily negative-emotion score and brain metabolism of the vmPFC/ACC cluster during the COVID-19 lockdown period (independently of age, sex, presence of focal morphological lesion, and duration of the lockdown; Pearson's correlation: *r* = −0.40, *p* = 0.00007).

Notably, the negative-emotion score was daily correlated with metabolism in the right ventromedial prefrontal cortex (vmPFC) and anterior cingulate cortex (ACC), with higher negative scores corresponding to lower metabolic activity in this region (peak T-score = 3.93, *p*-voxel < 0.001, *k* = 286, Figure 2A; Pearson's correlation: *r* = −0.40, *p* = 0.00007, Figure 3).

This relationship persisted after controlling for age, sex, the presence of focal lesions, or lockdown duration, as confirmed by partial correlation analysis (*r* = −0.39 to −0.41). ANOVA revealed no significant effect of PET scan indication (cognitive/behavioral impairment, glioma, or focal epilepsy) on vmPFC/ACC metabolism (*p* = 0.683).

**Figure 4:**
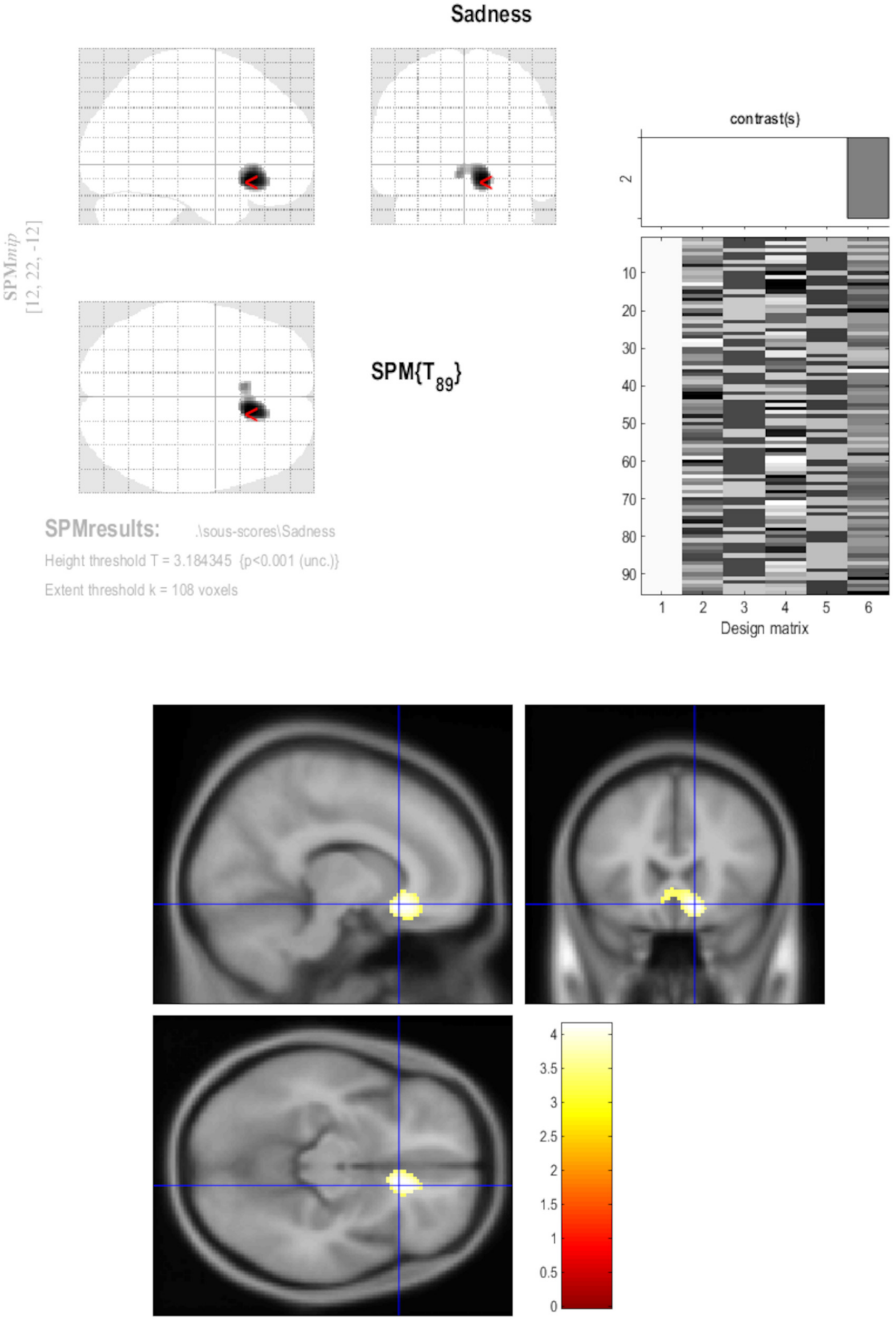
Brain PET T-maps relationships with the daily sadness score. A complementary voxel-wise analysis was performed for individual negative emotions, globally confirming the same vmPFC/ACC cluster, with a slightly larger bilateral cluster at right-side predominance for sadness (peak *T*-score; 4.13; *k* = 409).

Furthermore, the identified metabolic cluster showed similar negative correlations with individual negative emotions, including anger (*r* = −0.23, *p* = 0.02563), disgust (*r* = −0.28, *p* = 0.00516), fear (*r* = −0.32, *p* = 0.00144), sadness (*r* = −0.42, *p* = 0.00002), and surprise/amazement (*r* = −0.33, *p* = 0.00114). A complementary voxel-wise analysis was performed for individual negative emotions, confirming the same vmPFC/ACC cluster for fear, sadness (with a slightly larger bilateral cluster at right-side predominance: peak T-score = 4.13, *k* = 409; Figure 4), and surprise/amazement, while no significant findings were obtained at *p*-voxel < 0.001 for anger and disgust. No additional brain regions outside the vmPFC/ACC were identified.

Figure 2B illustrates the metabolic connectivity associated with the right vmPFC/ACC cluster, revealing a network predominantly involving the right hemisphere. This network primarily involves limbic and fronto-temporal regions, the insula, and the anterior cingulate cortex. Key structures within this network include the medial temporal lobe, encompassing the amygdala, hippocampus, and parahippocampus, as well as subcortical regions such as the basal ganglia, thalamus, and midbrain (Table 2).

**Table 2:** Metabolic connectivity analysis: anatomical regions connected to the vmPFC/ACC cluster, ranked by voxel extent (extracted using atlas from wfu_pickatlas: https://www.nitrc.org/projects/wfu_pickatlas/).

Voxels extent	Localization
7,320	Right Cerebrum
4,829	White Matter
3,474	Frontal Lobe
3,339	Gray Matter
2,831	Limbic Lobe
2,021	Sub-lobar
1,821	Left Cerebrum
1,541	Anterior Cingulate
1,105	Medial Frontal Gyrus
1,007	Sub-Gyral
931	Extra-Nuclear
893	Parahippocampa Gyrus
773	Hippocampus_R (aal3v1)
754	Temporal Lobe
686	Superior Frontal Gyrus
647	Rectus_R (aal3v1)
564	brodmann area 11
558	OFCmed_R (aal3v1)
527	ParaHippocampal_R (aal3v1)
495	Frontal_Med_Orb_R (aal3v1)
385	Inferior Frontal Gyrus
358	Inter-Hemispheric
353	Subcallosal Gyrus
349	brodmann area 25
348	brodmann area 10
329	Cerebro-Spinal Fluid
326	Frontal_Sup_Medial_R (aal3v1)
300	Frontal_Sup_2_R (aal3v1)
288	Lateral Ventricle
287	Thalamus
281	Caudate
274	brodmann area 24
272	ACC_sup_L (aal3v1)
266	Rectal Gyrus
265	ACC_sup_R (aal3v1)
265	brodmann area 32
265	Caudate_R (aal3v1)
261	ACC_pre_L (aal3v1)
254	Olfactory_L (aal3v1)
247	Caudate Head
227	Uncus
208	Frontal_Med_Orb_L (aal3v1)
203	Olfactory_R (aal3v1)
199	Corpus Callosum
185	Rectus_L (aal3v1)
175	brodmann area 47
171	Midbrain
168	Right Brainstem
163	ACC_sub_L (aal3v1)
162	OFCant_R (aal3v1)
157	Middle Frontal Gyrus
132	N_Acc_R (aal3v1)
131	Orbital Gyrus
128	ACC_sub_R (aal3v1)
127	Amygdala
121	Amygdala_R (aal3v1)
120	brodmann area 34
110	Lentiform Nucleus
108	OFCpost_R (aal3v1)
103	Hippocampus
95	Cingulate Gyrus
95	Fusiform_R (aal3v1)
90	Thal_VL_R (aal3v1)
86	N_Acc_L (aal3v1)
79	Superior Temporal Gyrus
70	ACC_pre_R (aal3v1)
64	Ventral Anterior Nucleus
63	brodmann area 28
55	Thal_VA_R (aal3v1)
53	Cingulate_Mid_L (aal3v1)
50	Pallidum_R (aal3v1)
48	Putamen
46	brodmann area 35
43	Lateral Globus Pallidus
40	Third Ventricle
38	Cerebellum Anterior Lobe
38	Culmen
38	Right Cerebellum
36	Thal_MDm_R (aal3v1)
36	Lingual_R (aal3v1)
35	Pulvinar
29	Insula
29	Temporal_Pole_Mid_R (aal3v1)
29	Temporal_Sup_R (aal3v1)
28	Frontal_Inf_Orb_2_R (aal3v1)
27	OFCpost_L (aal3v1)
26	Thal_LP_R (aal3v1)
25	ParaHippocampal_L (aal3v1)
25	Thal_AV_R (aal3v1)
25	Temporal_Pole_Sup_R (aal3v1)
24	Temporal_Mid_R (aal3v1)
24	Ventral Lateral Nucleus
24	Cerebellum_3_R (aal3v1)
24	brodmann area 33
22	brodmann area 27
21	Medial Dorsal Nucleus
21	Caudate Body
20	Insula_R (aal3v1)
20	Medial Globus Pallidus
19	Middle Temporal Gyrus
18	Thal_PuM_R (aal3v1)
18	brodmann area 21
18	Hypothalamus
16	Thal_MDl_R (aal3v1)
16	OFCmed_L (aal3v1)
13	Pallidum_L (aal3v1)
13	Thal_PuI_R (aal3v1)
13	Caudate Tail
12	brodmann area 13
12	Thal_IL_R (aal3v1)
10	Mammillary Body
9	Amygdala_L (aal3v1)
9	brodmann area 22
9	Red Nucleus
9	Cingulate_Mid_R (aal3v1)
8	Caudate_L (aal3v1)
8	SN_pr_R (aal3v1)
8	Lateral Dorsal Nucleus
7	Anterior Commissure
7	Cerebellum_4_5_R (aal3v1)
7	Frontal_Sup_Medial_L (aal3v1)
7	Thal_VPL_R (aal3v1)
7	Temporal_Pole_Sup_L (aal3v1)
6	Putamen_R (aal3v1)
6	brodmann area 38
6	Fusiform Gyrus
6	Optic Tract
5	Thal_PuL_R (aal3v1)
5	Putamen_L (aal3v1)
5	brodmann area 20
5	Thal_LGN_R (aal3v1)
5	brodmann area 30
5	Red_N_R (aal3v1)
4	brodmann area 36
4	Lateral Posterior Nucleus
4	Vermis_1_2 (aal3v1)
4	Midline Nucleus
2	Left Brainstem
2	Claustrum
2	Thal_PuA_R (aal3v1)
2	Temporal_Inf_R (aal3v1)
2	SN_pc_R (aal3v1)
2	Subthalamic Nucleus
2	Hippocampus_L (aal3v1)
1	Insula_L (aal3v1)
1	Thal_Re_R (aal3v1)
1	Substania Nigra
1	Vermis_3 (aal3v1)
1	Pons
1	VTA_R (aal3v1)

No overlap was observed between the right vmPFC/ACC cluster identified in this study and the previously reported hypometabolic patterns associated with physical deconditioning during the lockdown (6). Similarly, there was no overlap with the hypometabolic pattern observed in patients with long COVID (9). The metabolic network connected to the right vmPFC/ACC cluster demonstrated a 10.2% overlap with the long COVID hypometabolic pattern (9), while remaining fully distinct from the pattern linked to physical deconditioning.

## Discussion

The COVID-19 lockdown was an unprecedented global event that intensified negative emotions worldwide. By combining neuroimaging and sentiment analysis, our interdisciplinary approach connects subjective societal experiences to objective neurobiological changes, offering a perspective on how collective stress impacts brain function. Our findings demonstrate that collective negative emotions during the COVID-19 lockdown had measurable effects on brain function, offering a potential neurobiological explanation for the increased psychological and psychiatric disorders reported during the pandemic. Beyond the COVID-19 pandemic, such innovative framework holds potential for extension to other contexts of collective stress.

As a proxy for psychological assessment, we utilized a composite negative-emotion score derived from sentiment analysis of a large open-source French X/Twitter dataset, previously published, designed to capture global public sentiment during the 55-day lockdown (15). We then investigated the relationship between this psychological proxy and brain PET metabolism in patients with neurological conditions. By focusing on patients with neurological disorders, this study aimed at shedding light on the heightened vulnerability of this population to societal stressors. Alterations were identified in the right ventromedial prefrontal cortex (vmPFC) and anterior cingulate cortex (ACC), as well as in connected regions. This connectivity underscores the involvement of a broad, limbic-dominant neural circuit, especially the right amygdala, forming a critical network for mental health, emotion regulation, and responses to fear and stress. Correlations found for emotions such as fear, sadness, and amazement confirm their key role in affecting fear circuits during collective stress. These more specific, convergent findings suggest a consistent association between negative emotional states and reduced metabolic activity in the right vmPFC/ACC. While statistically significant, this association is moderate in strength (*r* = −0.40) and aligns with normative expectations in population neuroscience (14), where small to moderate effect sizes are commonly reported at the group level when investigating brain–environment interactions. Notably, the metabolic patterns identified in this study differ from those linked to physical deconditioning during the lockdown (6) or long COVID (9), suggesting the involvement of additional—and potentially cumulative—mechanisms. The limited overlap between these patterns could also be influenced by patients' history of COVID-19, although this information was not individually available for this study population.

On the whole, these findings could play a pivotal role in informing public policies aimed at anticipating and mitigating the psychological impacts of pandemics and other global crises. More specifically, the results highlight the need for targeted mental health interventions to support at-risk groups during periods of collective stress, ensuring that vulnerable populations receive the care and resources they need to navigate such challenges effectively.

### Mental health pandemic issues

There is growing evidence of the negative impact of pandemics on cognitive and mental functioning (1, 2). Psychological and psychiatric disorders have been particularly prevalent and persistent among at-risk populations, including children and adolescents (24), students (25), the elderly (26), and healthcare professionals (27). Key factors influencing the likelihood and severity of these mental health issues encompass the duration of the pandemic, the severity of illness, and pre-existing mental health conditions. The prolonged exposure of millions to stressors such as fear, uncertainty, and isolation—factors that profoundly disrupt mental well-being (28)—are known to modulate the brain's fear circuitry. This triggers neural pathways that prime the body for fight-or-flight responses, leading to a heightened state of arousal. Crucially, processes such as fear conditioning and extinction play a central role in the development and persistence of threat-related disorders. Over time, the arousal state can contribute to symptoms of PTSD and increased anxiety. These insights highlight the profound neuropsychiatric consequences of pandemics and underscore the need for targeted interventions to mitigate their long-term effects.

### The neurobiological substrate

From a neurobiological perspective, the experience of fear emerges from the intricate interplay of various brain regions that form complex neural networks implicated in emotional disorders. While the amygdala is often regarded as the brain's “fear center” due to its critical role in processing emotional stimuli and initiating fear responses, the fear network extends beyond the amygdala to include other key regions. This study highlights the ventromedial prefrontal cortex (vmPFC) and anterior cingulate cortex (ACC) as crucial components of this network (29). The ACC plays a central role in monitoring conflicts, detecting errors, and processing emotional responses (30), as well as in neural circuit of placebo effect (31), while the vmPFC is associated with higher-order cognitive functions, including decision-making and emotional regulation (32). When a fearful stimulus is detected, the amygdala activates physiological responses, such as increased heart rate and sweating. Simultaneously, the ACC evaluates the threat's significance and potential consequences, while the vmPFC modulates the fear response by suppressing excessive fear and promoting adaptive behaviors. In essence, the fear network operates through a coordinated interaction: the amygdala detects threats, the ACC assesses their importance, and the vmPFC regulates the emotional response to ensure an adaptive outcome (33).

The findings of this study, which demonstrate that increased negative-emotion scores are associated with decreased resting-state metabolism in the vmPFC/ACC, align with previous research. Hypometabolism in these regions has been observed in PTSD patients using [18F]FDG PET imaging (34), often alongside MRI-detected atrophy (35), as well as in generalized social anxiety disorder again using [18F]FDG PET imaging (36). In PTSD, hypoactivity in the vmPFC and hippocampus is associated with an impaired ability to consolidate extinction learning (37), which prevents patients from recognizing that they are no longer in a state of danger. The observed decrease in vmPFC metabolic activity during the French COVID-19 lockdown, linked to heightened negative emotions, likely reflects similar difficulties in emotion regulation and adaptation. Moreover, the PTSD impairment extends beyond the vmPFC to the broader PTSD threat circuit, including the ACC, amygdala, and hippocampus (38), as shown in this study. These results suggest that the psychological stress of the French COVID-19 lockdown engaged the same neuronal mechanisms typically associated with post-traumatic events, further emphasizing the profound neurobiological impact of collective emotional stress during this period.

### Perspectives

Negative emotions and collective stress affect brain function, underscoring the critical need for proactive strategies to mitigate the mental health consequences of future crises. Policies aimed at supporting mental health and targeted communication campaigns are essential tools to reduce the psychological burden, particularly among vulnerable groups such as individuals with neurological and psychiatric conditions, isolated individuals, and frontline workers. Initiatives that promote positive and reassuring information, ensure transparency, counter misinformation, and strengthen community support networks can be instrumental in shaping collective emotional resilience during crises. By combining optimism with transparency, such communication builds trust and supports adaptive responses to challenges. This approach aligns with principles of positive psychology, which emphasize that fostering hope and focusing on actionable solutions can mitigate the psychological burden of crises. Furthermore, it resonates with recent insights into the neurobiological mechanisms of the placebo effect, which conversely involve the activation of the rostral ACC (31). Without denying difficulties, resilience emerges by empowering individuals and communities to overcome them with confidence, grounded in evidence-based knowledge (39–42). In this sense, “thinking good”—when supported by truth and constructive action—not only fosters recovery but also lays the foundation for “it to be good” in the collective experience.

From a clinical perspective, these findings highlight the potential of identifying biomarkers as valuable tools for assessing individual vulnerability to collective stress. [18F]FDG-PET, beyond its possible clinical application during viral pandemics to detect inflammatory or infectious complications (19), also offers a promising avenue for investigating cerebral alterations associated with societal stress. Such biomarkers could guide the development of tailored therapeutic interventions, including personalized cognitive-behavioral therapies, stress management programs, robust community-based support systems, and next advanced brain stimulation techniques. These approaches have the potential to enhance resilience, improve emotional regulation, and mitigate the long-term psychological impacts of collective stressors, thereby paving the way for more precise and effective mental health care strategies.

Additionally, the methodological framework of this study could be adapted to other crises, such as wars, climate-related disasters, or social conflicts. Expanding its application would provide deeper insights into the broader impacts of collective emotional stress on brain function, facilitating the development of universal strategies to address the neurobiological and psychological challenges posed by major societal disruptions.

### Limitations

Despite the relatively large sample size, this study is constrained by several limitations. Its retrospective design precludes the inclusion of detailed clinical evaluations of daily activities, cognitive function, or mental health, and it lacks longitudinal follow-up. The findings are also restricted to patients with neurological disorders, who represent a population particularly vulnerable to collective stress and negative emotions due to their pre-existing conditions. Neurological alterations in these individuals could indeed amplify the sensitivity of brain circuits, such as the vmPFC and ACC, making them more susceptible to stressful contexts like confinement and negative emotion. Consequently, the results may reflect a specific fragility of this population, characterized by reduced resilience to stress due to deficits in emotional regulation, and may not be generalizable to individuals without neurological disorders. Beyond this increased vulnerability, it is also relevant to consider how specific cerebral conditions may interact with the fear network. For instance, gliomas, depending on their frequent localization and peritumoral effects within the insular region, may disrupt the connectivity between prefrontal and limbic regions, altering emotional processing and regulation (43). Similarly, patients with focal epilepsy often present with altered limbic connectivity, particularly in the amygdala and hippocampus, regions central to the fear response (44). Seizure-related neuroinflammation and hippocampal sclerosis could further modulate the activity of the fear circuit. Additionally, patients with cognitive or behavioral impairments, such as those with neurodegenerative diseases (45), frequently exhibit vmPFC and ACC dysfunctions, which are key nodes for emotion regulation. Such dysfunctions could contribute to exaggerated responses to negative emotional stimuli or impair adaptive coping mechanisms. Finally, morphological lesions may cause disconnection effects between the vmPFC, ACC, and subcortical structures (e.g., thalamus, basal ganglia), altering the dynamic regulation of fear responses. While our study controlled the presence of focal lesions, their potential influence on network connectivity should be considered. Future studies using multimodal imaging approaches, such as diffusion tensor imaging (DTI) or functional MRI (fMRI), could provide further insights into how these neurological conditions shape the response of the fear network under collective stress conditions.

Although factors such as age and sex were controlled, other contextual variables — such as socioeconomic status, family dynamics, degree of exposure to COVID-19, pathological characteristics (including specific diagnosis for cognitive/behavioral impairment, neuropsychological evaluations), and psychiatric comorbidities — could influence the findings. Moreover, as this is an observational study, the identified correlations between negative emotions and metabolic changes do not establish causality. Experimental studies would be necessary to directly investigate these mechanisms. Additionally, the observed functional metabolic effects are not synonymous with structural damage or irreversible changes.

The study also acknowledges limitations in the use of X/Twitter data, as the emotions expressed on this platform may not accurately represent those of the general population. X/Twitter users tend to differ from the broader public in terms of demographics, socioeconomic status, and emotional expression. Expanding data sources to include longitudinal surveys, personal diaries, or other social networks could provide a more comprehensive view of collective emotional states.

Future research should include longitudinal studies to determine whether the observed metabolic changes persist over time and whether they predict long-term psychological or neurological outcomes, such as PTSD, anxiety disorders, or even the maintenance of an overall negative emotional attitude.

This study bridges neuroscience and societal analysis to investigate the brain PET metabolic changes in response to the psychological stress experienced during the COVID-19 lockdown. While our long-term goal would be to understand broader societal emotional responses to crises, this study specifically focuses on patients with neurological disorders, who may be as mentioned particularly vulnerable to societal stressors. Expanding the study to healthy individuals and diverse stress contexts—such as climate disasters, wars, or social conflicts—could broaden the applicability of these findings. Multicenter studies across different countries and cultures are essential to validate these results and establish the universality of the brain metabolic changes in response to collective emotional stress.

## Conclusion

This study highlights the impact of societal emotional stress on brain metabolism, particularly in neurologically vulnerable population during the COVID-19 lockdown. Our findings emphasize the need for tailored mental health interventions for at-risk groups. Moreover, they underscore the critical role of contextual factors in shaping collective emotional responses and their neurobiological consequences. Expanding this research to include broader populations and longitudinal analyses could provide a deeper understanding of these mechanisms, ultimately to inform strategies mitigating the neurological and psychological effects of future global crises.

## Data Availability

Publicly available datasets were analyzed in this study. This data can be found here: Balech S, Benavent C, Calciu M. The First French COVID19 Lockdown Twitter Dataset. Published online 2020, https://arxiv.org/abs/2005.05075.
