# Correction: Emotional stress during the COVID-19 lockdown: how negative X/Twitter posts correlated with changes in the brain’s fear network

**DOI:** 10.3389/fnume.2025.1655239

**Published:** 2025-07-10

**Authors:** Eric Guedj, Jacques-Yves Campion, Tatiana Horowitz, Fanny Barthélémy, Stéphanie Khalfa, Wissam El-Hage

**Affiliations:** ^1^ Aix Marseille Univ, APHM, CNRS, Centrale Marseille, Institut Fresnel, Timone Hospital, CERIMED, Nuclear Medicine Department, Marseille, France; ^2^ Université de Tours, INSERM, Imaging Brain & Neuropsychiatry IBraiN U1253, Tours, France; ^3^ Department of Psychiatry, University of Pittsburgh, Pittsburgh, PA, United States; ^4^ Aix Marseille Univ, APHM, CNR, Centre de Recherches en Psychologie et Neurosciences, Marseille, France; ^5^ CHRU de Tours, Regional Trauma Center CRP-CVL, Tours, France

**Keywords:** COVID-19, SARS-CoV-2, lockdown, [18F]FDG PET, brain metabolism, Twitter, media, communication

Correction on Emotional stress during the COVID-19 lockdown: how negative X/Twitter posts correlated with changes in the brain's fear network By Guedj E, Campion JY, Horowitz T, Barthélémy F, Khalfa S, El-Hage W (2025). Front Nucl Med. 5:1575026. doi: 10.3389/fnume.2025.1575026

**Figure/table caption**

There was a mistake in the caption and the *x*-axis label of Figure 1 as published.

The caption was displayed as “Distribution of composite negative-emotion scores by day of lockdown”.

The corrected caption of Figure 1 appears below.

“Distribution of composite negative-emotion scores across patients”

The *x*-axis label was displayed as “#day of lockdown”.

The corrected *x*-axis label of Figure 1 appears below.

“#patient”

The original version of this article has been updated.

